# Learning to Perceive Non-Native Tones via Distributional Training: Effects of Task and Acoustic Cue Weighting

**DOI:** 10.3390/brainsci12050559

**Published:** 2022-04-27

**Authors:** Liquan Liu, Chi Yuan, Jia Hoong Ong, Alba Tuninetti, Mark Antoniou, Anne Cutler, Paola Escudero

**Affiliations:** 1 The MARCS Institute for Brain, Behaviour and Development, Western Sydney University, Westmead, NSW 2145, Australia; c.yuan@hfut.edu.cn (C.Y.); jiahoong.ong@reading.ac.uk (J.H.O.); alba.tuninetti@bilkent.edu.tr (A.T.); m.antoniou@westernsydney.edu.au (M.A.); a.cutler@westernsydney.edu.au (A.C.); paola.escudero@westernsydney.edu.au (P.E.); 2 School of Psychology, Western Sydney University, Westmead, NSW 2145, Australia; 3 Center of Multilingualism across the Lifespan, University of Oslo, 0316 Oslo, Norway; 4 Australian Research Council Centre of Excellence for the Dynamics of Language, Canberra, ACT 2601, Australia; 5 School of Foreign Studies, Hefei University of Technology, Hefei 230009, China; 6 School of Psychology and Clinical Language Sciences, University of Reading, Reading RG6 6AH, UK; 7 Department of Psychology, Bilkent University, Ankara 06800, Turkey

**Keywords:** distributional learning, tone, discrimination, identification, oddball-EEG, phonetic distance, acoustic cue-weighting

## Abstract

As many distributional learning (DL) studies have shown, adult listeners can achieve discrimination of a difficult non-native contrast after a short repetitive exposure to tokens falling at the extremes of that contrast. Such studies have shown using behavioural methods that a short distributional training can induce perceptual learning of vowel and consonant contrasts. However, much less is known about the neurological correlates of DL, and few studies have examined non-native lexical tone contrasts. Here, Australian-English speakers underwent DL training on a Mandarin tone contrast using behavioural (discrimination, identification) and neural (oddball-EEG) tasks, with listeners hearing either a bimodal or a unimodal distribution. Behavioural results show that listeners learned to discriminate tones after both unimodal and bimodal training; while EEG responses revealed more learning for listeners exposed to the bimodal distribution. Thus, perceptual learning through exposure to brief sound distributions (a) extends to non-native tonal contrasts, and (b) is sensitive to task, phonetic distance, and acoustic cue-weighting. Our findings have implications for models of how auditory and phonetic constraints influence speech learning.

## 1. Introduction

Listening to speech involves identifying linguistic structures in a fast, continuous utterance stream and processing their relative ordering to retrieve an intended meaning. In the native language, multiple sources of relevant information are drawn upon to accomplish this task, including knowledge of the vocabulary and the relative likelihood of different sequential patterns at lower and higher levels of linguistic structure, as well as the rules governing sequential processes at the phonetic level. Non-native languages can differ from a listener’s native languages on all these dimensions. We here address the question of whether listeners faced with the task of discriminating a novel non-native contrast show preferences as to which dimension of linguistic information they attend to (or attend to most). To better understand the source of any such preference, we compare behavioural against neurophysiological measures.

There is a considerable literature concerning the perception and learning of non-native contrasts, and it naturally shares ground with the very extensive literature on native contrast learning, with the differences in large part arising from learner constraints, given that in the native situation, the learners are still in infancy. Interestingly, overlap between the native and non-native literatures has been increased by the growing attention paid to statistical accounts of learning processes, due to the extensive evidence showing that such information is not restricted to mature brains, but is processed even by the youngest of learners.

Moreover, statistical learning is not specific to the language domain. It is observed for visual objects [1], spatial structures [2], tactile sequences [3] and music perception [4]. Crucially, two-month-olds exhibit neural sensitivity to statistical properties of non-native speech sounds during sleep [5], indicating that statistical learning is a formative mechanism that may have considerable explanatory power for our understanding of perceptual learning processes.

The language acquisition process can draw on statistical regularities in the linguistic environment ranging from simple frequency counts to complex conditional probabilities, including transitional probabilities [6], non-adjacent dependencies of word occurrences [7] and distributional patterns of speech sounds [8]. Listeners’ most basic task, namely segmenting continuous speech streams into words and speech sounds, can also be assisted by attention to the distributional properties of the input [9]. Even infants in their first year attend to such information. Maye and colleagues [8] showed that infants as young as 6 months of age use distributional properties to learn phonetic categories. They exposed infants to manipulated frequency distributions of speech sounds that differed in their number of peaks, producing typically unimodal (one-peak) versus bimodal (two-peak) distributions, with the former thought to support a single, large category, while the latter (a distribution with two peaks) promotes the assumption of two categories.

A major factor affecting distributional learning is age, with learning appearing to be more effective for infants than for adults [10] and for younger than older infants [11]. Another factor is attention, with better results for learners whose task requires them to attend to the stimuli, as opposed to those who do not need to payattention to the stimuli’s properties [12]. A third factor is the amount of exposure provided to the learner, with more extended distributional exposure yielding better discrimination results [13]. Fourth, experimental design also plays a role: A learning effect is more likely to appear with a habituation-dishabituation procedure than with familiarization-alternation paradigms [14]. Fifth and not least, the learning target itself can directly impact learning outcomes [15] due to its acoustic properties (a non-native contrast similar to the native inventory is more rapidly acquired through DL than a dissimilar contrast [16]) or its perceptual salience (consonants are relatively less salient acoustically than vowels and are thus less rapidly learned distributionally [17,18]). The last factor further leads to a series of cue-weighting studies demonstrating that statistical information in the ambient environment is not the only cue listeners adopt. For example, when learning a speech sound contrast, learners may focus more on the phonetic/acoustic properties of stimuli than statistical regularities constraining the contrast [19,20,21,22].

Most studies in this literature so far have focused on consonants and vowels. But speech has further dimensions, including suprasegmental structure which is manifested on many levels, including the lexical level. Around 60–70% of the world’s languages are tonal, i.e., they use pitch to distinguish word meanings [23]. Tone language speakers show categorical perception for tones, but non-tone language speakers report them as psycho-acoustic rather than linguistic [24]. However, acoustic interactions of tonal and segmental information affect simple discrimination task performance equivalently for native listeners of a tone language and non-native listeners without tonal experience [25]. Specifically, both Cantonese and Dutch listeners take longer and are less accurate when judging syllables that differ in tones compared to segments, suggesting a slower processing of tonal than segmental distinctions, at least in speeded-response tasks.

Non-tonal language speakers have substantial difficulty discriminating and learning tone categories [26], and studies have shown mixed results after distributional training [27]. For infants, bimodal distributional training enhances Mandarin tone perception for Dutch 11-month-olds, but not for 5- or 14-month-olds [28]. For adults, Ong and colleagues [12] found that Australian-English speakers exposed to a bimodal distribution of a Thai tone contrast showed no automatic learning effect; but when the training involved active listening (through a request for acknowledging the heard stimuli), their sensitivity to tone improved. Interestingly, exposure to a bimodal distribution of Thai tones resulted in enhanced perception of both linguistic and musical tones for Australian-English speakers, demonstrating cross-domain transfer [4,29]. Note that linguistic and musical tone perception also tend to be correlated in non-tone language adults’ performance [26,30] and in psycho-acoustic perception [31].

All the above results stem from behavioural research because statistical learning research so far has made little use of neurophysiological methods, despite evidence that neural responses can provide processing information at both pre-attentive and cortical levels [32,33]. Compared to behavioural measures, however, neural techniques such as electroencephalography (EEG) have the benefit of requiring no overt attention or decision processes. They are typically sensitive to early pre-attentive responses, reflecting the neural basis of acoustic-phonetic processing [34,35] and providing another measure of implicit discrimination.

Importantly, evidence from non-native speech perception studies shows that non-native listeners exhibit mismatch negativity (MMN) responses for contrasts they do not discriminate in behavioural tasks [36,37]. The MMN response has been used extensively in speech perception studies to examine how the perceptual system indexes incoming acoustic stimuli pre-attentively, i.e., in a manner not requiring overt attention. Based on the formation of memory traces, the MMN response is a negative-going waveform that is typically observed in the frontal electrodes. It signals detection of change in a stream of auditory stimuli, and specifically indicates differences between stimuli with different acoustics. It is obtained by computing the difference in the event-related potential (ERP) response to an infrequent (termed deviant) stimulus versus a frequent (termed standard) one. This response typically occurs 150 to 250 ms post onset of the stimulus switch [38,39].

Despite the scarcity of neurally-based research on distributional learning, some evidence is available on listeners’ perceptual flexibility for the tonal dimension of speech. In tone perception, pitch height and pitch direction are the two main acoustic cues [40]. Nixon and colleagues [41] explored German listeners’ neural discrimination of a Cantonese high-mid pitch height contrast, by exposing them to a bimodal distribution of a 13-step tone continuum. Although a prediction of enhanced MMN responses at the two Gaussian peaks was not supported, the listeners’ perception of pitch height improved across all steps along the continuum. A follow-up study of listeners’ neural sensitivity to cross-boundary differences again showed enhanced sensitivity to overall pitch differences over the course of the tone exposure [42]. In other words, acuity with respect to acoustic pitch differences was increased during distributional learning not only between but also within categories. In contrast to the findings of previous studies testing segmental features, these results indicate that exposure to a bimodal distribution may not necessarily lead to the enhanced discrimination of specific steps or categories along the pitch continuum, but may rather alter listeners’ overall sensitivity to tonal changes. A caveat in this interpretation is that these studies did not include a unimodal distribution. Taken together, when EEG studies are adopted to examine tone perception and learning, results often illustrate robust sensitivity not merely restricted to the patterns predicted by frequency distributions as shown in behavioural studies.

On the same note, a recent study examining 5–6-month-old infants’ neural sensitivity to an 8-step contrast of flat versus falling pitch (a Mandarin tone contrast) found a surprising enhancement effect after exposure to a unimodal but not a bimodal distribution [43]. This finding was explained in relation to listeners’ acoustic sensitivity to frequently heard tokens at peak locations along the tone continuum. The high-frequency tokens had smaller differences in the unimodal (steps 4–5) than in the bimodal (steps 2–7) distributions. The authors of [43] argued that frequent exposure to greater acoustic distance may lead to reduced neural sensitivity to a smaller acoustic distance (steps 3–6). This interpretation highlights the role of the magnitude of the acoustic distinctions in the stimuli when prior training and exposure is insufficient to establish phonetic categories, which can be explained by models of non-native perception that focus on acoustic cue weighting and salience (see for instance, [44]).

In the present study, we examined tone perception and learning for non-tonal language speakers, collecting and directly comparing behavioural and neural responses. Australian English listeners with no prior knowledge of any tone language heard a Mandarin tone contrast. Tone perception ability has long been known to vary as a function of the acoustic properties of the tonal input [45]. We varied tone features and the nature of the input distribution (comparing uni- versus bimodality). Experiment 1 tested listeners’ ability to discern the level (T1)–falling (T4) Mandarin tone contrast before and after distributional training, using discrimination and identification tasks, respectively. Experiment 2 then examined listeners’ neural sensitivity to the same contrast, in a standard MMN paradigm, assessing amplitude, latency, anteriority, laterality and topographic differences between the two modalities. Previous work has shown differences in factors such as anteriority and laterality depending on stimulus characteristics and participant language background [46,47,48] while other work has not [33,49]. We included them here to ensure that we captured the most comprehensive set of results for examining potential effects of distributional learning at the neural level.

## 2. Experiment 1: Mandarin Tone Discrimination and Identification by Australian Listeners before and after Distributional Learning

### 2.1. Methods

#### 2.1.1. Participants

Forty-eight native Australian English speakers naïve to tone languages took part (36 females; *M*_age_ = 23.06, *SD*_age_ = 5.57). Nine participants reported having musical training (ranging from 1 to 10 years), though only one continued to practise music at the time of testing. All participants reported normal speech and hearing, provided written informed consent prior to testing, and received course credit or a small monetary compensation.

#### 2.1.2. Stimuli

**Figure 1:**
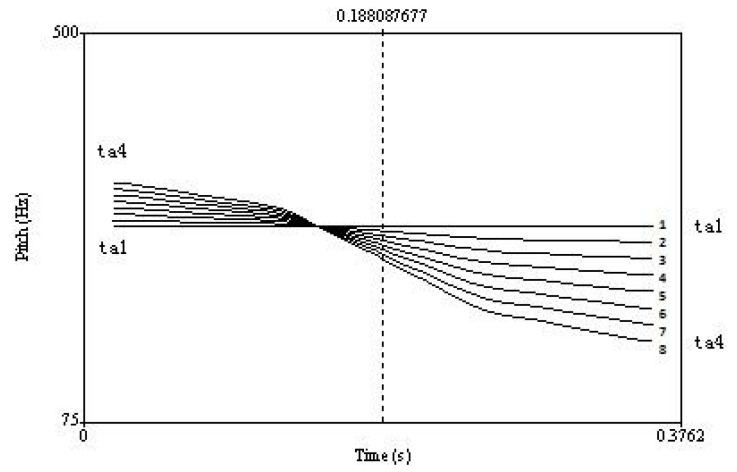
Pitch contours along a /taT1/–/taT4/ continuum (Stimuli and figure from [28]).

This study focused on the Mandarin Chinese level [T1] versus falling [T4] tonal contrast. A female Mandarin speaker produced natural tokens of /taT1/ (‘take’) and /taT4/ (‘big’) in a soundproof booth. Recording used the computer program Audacity and a Genelec 1029A microphone, with a 16-bit sampling depth and a sampling rate of 44.1 kHz. To create the 8-step continuum, equidistant stimulus steps differing in pitch contour were constructed from /taT1/ to /taT4/ (Figure 1) using the following procedure in Praat [50]: First, four interpolation points along the pitch contours (at 0%, 33%, 67% and 100%) were marked (Supplementary Material A). Next, the distances (in Hz) between the corresponding points were divided into seven equal spaces, generating six new layers. New pitch tokens were then created by connecting the four corresponding intermediate points on the same layer. This formed a continuum of eight steps (including the endpoint contours) from /taT1/ (step 1) to /taT4/ (step 8). Stimulus intensity was set to 65 dB SPL and duration was set to approximately 400 ms. Five native Mandarin speakers listened to the stimuli and confirmed that they were acceptable tokens of these Mandarin syllables. The contrast (with the same stimuli) has been used in previous studies [51,52,53,54].

#### 2.1.3. Procedure

The experiment consisted of three phases in the following order: pre-test, distributional learning and post-test. Task programming, presentation of stimuli and response recording were conducted using E-Prime (Psychology Software Tools Inc., Sharpsburg, PA, USA) on a Dell Latitude E5550 laptop. Auditory stimuli were presented at 65 dB SPL via Sennheiser HD 280 Pro headphones and were ordered randomly. No corrective feedback was given. The experiment took approximately 40 min to complete.

**Figure 2:**
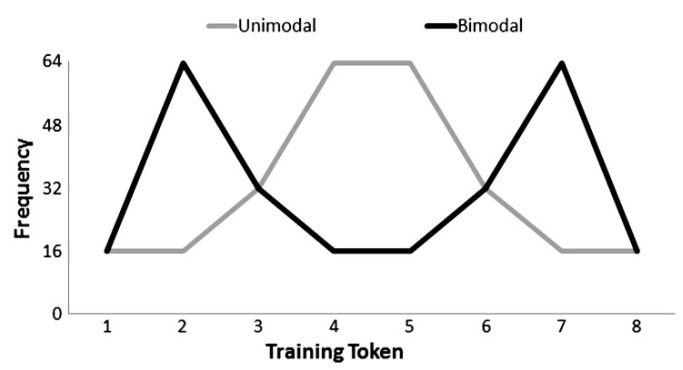
Frequency of occurrence for each training token encountered by listeners in the unimodal (grey line) and bimodal (black line) conditions. Figure from [55].

In the distributional learning phase, participants were randomly assigned to one of the two conditions: unimodal or bimodal distribution (Figure 2). The two conditions had different distributional peaks (a single central category vs. two separate categories) but were equal in terms of the total amount of distributional learning tonal input (256 tokens) and duration (360 s). In the bimodal condition, stimuli from the peripheral positions of the continuum were presented with higher frequency. In other words, participants heard tokens of steps 2 and 7 most frequently. In the unimodal condition, stimuli near the central positions, namely tokens of steps 4 and 5, were presented most frequently. Crucially, stimulus steps 3 and 6 were presented an equal number of times in both conditions.

At pre- and at post-test, participants were first presented with a discrimination task in which they indicated via keypresses whether paired lexical tone stimuli steps were the same or different. Trials included same (e.g., steps 1–1, 2–2) and different pairs (e.g., steps 2–4, 3–6) along the continuum, with each pair presented 10 times. Trials not involving the target contrast functioned as controls. The inter-stimulus interval between tokens was 1000 ms.

Participants then completed an identification task in which they indicated (again via keypresses) whether the tone of each continuum step (e.g., step 3) was a flat tone (indicated by a flat arrow) or a falling tone (indicated by a falling arrow), with each tone presented 6 times. For each task, four auditory examples were played as practice trials prior to testing. Trials were self-paced and presented in random order.

Analyses for the discrimination task targeted the perception of steps 3 to 6, and those for the identification task focused on step 3 and step 6. As an additional control, the Pitch-Contour Perception Test (PCPT; [56,57]) was included in the post-training phase, to examine participants’ pitch perceptual abilities (Supplementary Material B). This test required indication of whether isolated tone tokens had a flat, rising or falling contour. The PCPT allows allocation -of participants to high and low aptitude groups, to examine whether ability to perceive pitch affects identification and discrimination responses. No differences either in the identification or in the discrimination tasks were observed between listeners with high versus low aptitude in the present study.

### 2.2. Results

**Figure 3:**
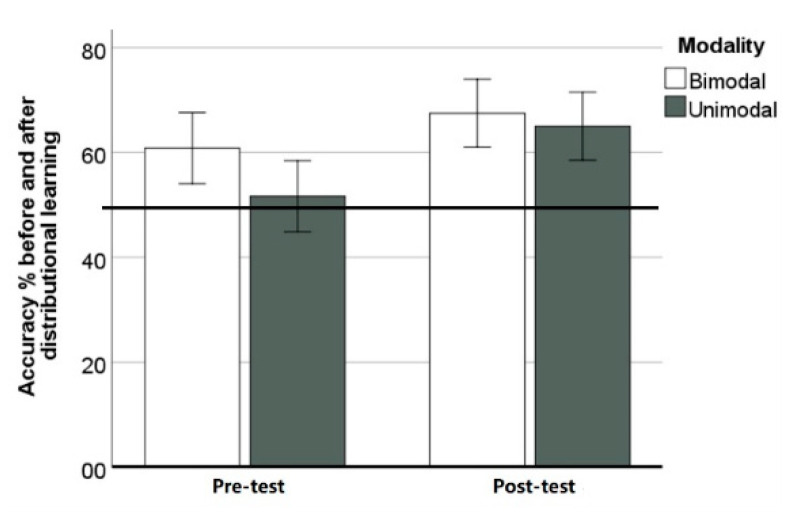
Mean accuracy percentage before and after distributional learning (Error bars = ±1 standard error). The horizontal line indicates chance level (50%) performance.

We first compared listeners’ percentage of accurate choices for the target contrast (steps 3–6) in the discrimination task before and after distributional learning to chance (50%) with a one-sample t-test (Table 1). Neither condition showed discrimination above chance before distributional learning, while after training, listeners in both conditions were able to discriminate the contrast. We then conducted a Repeated Measures Analysis of Variance (RM ANOVA) with condition (2-level, unimodal vs. bimodal) as the between-subjects factor and accuracy (2-level, pre- and post-training phases) as the within-subjects variable (Figure 3). The main effect of training was significant (*F*(1, 46) = 4.731, *p* = 0.035, *η*_g_^2^ = 0.093), indicating a difference in accuracy before and after distributional learning. The interaction between condition and test phase was not significant (*F*(1, 46) = 0.526, *p* = 0.472, *η*_g_^2^ = 0.011), suggesting no difference between unimodal and bimodal exposure. In other words, listeners’ tone discrimination improved after exposure to either distribution.

**Figure 4:**
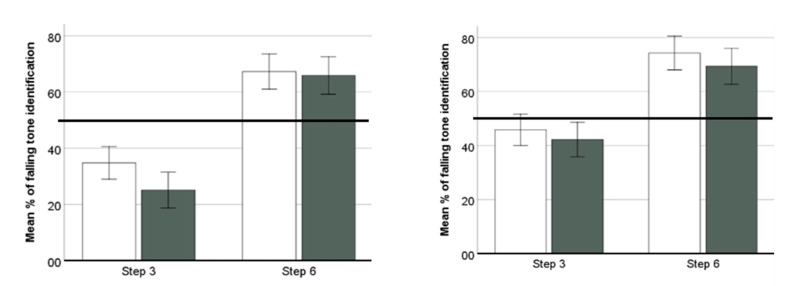
Mean percentage of “falling” classifications for steps 3 and 6 before and after bimodal (**left**) and unimodal (**right**) distributional learning (Error bars = ±1 standard error) White bars indicate performance at pretest, and black bars posttest. The horizontal line indicates chance (50%) performance.

We then examined listeners’ choices in the identification task before and after learning (Table 2). Across participants, Step 6 was more often identified as falling than step 3. We then conducted a repeated measures ANOVA with condition (2-level, unimodal vs. bimodal) as the between-subjects factor, and with percentage of falling choices across phase (2-level, pre- and post-training) and step (2-level, steps 3 & 6) as the within-subjects variables (Figure 4). The only significant factor was step (*F*(1, 46) = 57.343, *p* < 0.001, *η*_g_^2^ = 0.555). No other factors or interactions were significant (*F*s < 1.936, *p*s > 0.170, *η*_g_^2^ < 0.040). In contrast to the discrimination outcomes, no trace of improvement was observed in listeners’ tone identification after either distributional condition.

### 2.3. Discussion

Experiment 1 used behavioural measures to investigate how distributional learning of a Mandarin tone contrast affects listeners’ tone discrimination and identification. Listeners showed improved discrimination abilities after exposure to either unimodal or bimodal distributions, and no change was observed in their identification patterns.

In the discrimination task, successful distributional learning would predict enhanced discrimination of steps 3–6 after the bimodal condition, and/or reduced discrimination of this step after the unimodal experience. However, our results showed that listeners’ tonal sensitivity was enhanced after distributional learning irrespective of the embedded statistical information. Statistical exposure appears to benefit participants more in acoustic than in statistical cues. This interpretation resembles reported EEG studies with a Cantonese tone contrast [41,42], and agrees with findings showing that listeners attend more to prosodic than statistical cues to segment speech streams [20]. It is worth mentioning that only a single (bimodal) distribution was tested in these cited studies.

In identification, listeners’ difficulty in anchoring non-native tones to given categories plausibly reflects their lack of tonal categories in the first place (N.B. performance differences in identification versus discrimination have been attested before.) Non-tone language speakers often find it hard to make recognition responses to tones. Their much better tone discrimination ability, in contrast, reflects sensitivity to general pitch information (note that pitch perception in language and music correlate [26,30]).

To further examine the relationship between tone processing, distributional learning, and the cues listeners pay attention to in these processes, Experiment 2 examined listeners’ neural changes induced by distributional learning.

## 3. Experiment 2: Australian Listeners’ Neural Sensitivity to Tones before and after Distributional Learning

### 3.1. Methods

#### 3.1.1. Participants

A new sample of 32 Australian English speakers naïve to tone languages participated in Experiment 2 (22 females; *M*_age_ = 22.9, *SD*_age_ = 7.60). Eight participants reported having musical training (ranging from 1 to 7 years), though only one continued to practise music at time of test. Participants provided written informed consent prior to the experiment and received course credit or a small reimbursement for taking part.

#### 3.1.2. Stimuli

The same stimuli were used as in Experiment 1, except that the duration of all stimulus tokens was reduced to 100 ms to accommodate to the EEG paradigm.

#### 3.1.3. Procedure

As in Experiment 1, there were three phases: pre-test, distributional learning, and post-test. In the distributional learning phase, participants were randomly assigned to either the unimodal or the bimodal condition, with equal numbers of bilingual and monolingual participants in each condition. The two conditions did not differ in the total number of exposure trials (256 tokens) or duration (360 s) but varied in the frequency distribution (one vs. two Gaussian peaks) along the phonetic continuum only. Stimuli near the central positions were presented most frequently in the unimodal condition, whereas those from the peripheral sides of the continuum were presented with the highest frequency in the bimodal condition. Importantly, the frequency of occurrence of tokens from steps 3 and 6 was again identical across both conditions.

An EEG passive oddball paradigm was used for pre- and post-test, in which two separate blocks were presented. Step 3 was standard and step 6 was deviant in one block, and this was reversed in the other. The standard-deviant probability ratio was 80–20%. Since steps 3 and 6 were presented the same number of times in each condition, any potential differences observed in the post-test should be attributed to the condition. The sequence of the blocks was counterbalanced. No fewer than three and no more than eight standard stimuli occurred between deviant stimuli. Each block started with 20 standards and contained 500 trials in total. The inter-stimulus interval was randomly varied between 600 and 700 ms. Following the presentation phase, participants heard as a control (lasting approximately 1 min) 100 instances of the deviant stimuli they had heard in the previous oddball presentation. This design allowed a response comparison of the same number of deviant stimuli in the oddball presentation (*N* = 100) and the control presentation (*N* = 100).

Participants were tested in a single session in a sound-attenuated booth at the MARCS Institute for Brain, Behaviour and Development at Western Sydney University. They watched a self-selected silent movie with subtitles during the experiment and were instructed to avoid excessive motor movement and to disregard the auditory stimuli. The stimuli were presented binaurally via Etymotic earphones with the intensity set at 70 dB SPL in Praat [50] and the volume level was set at a comfortable listening level consistent across participants as a result of piloting that showed MMN elicitation. The duration of the EEG experiment was approximately 45 min.

#### 3.1.4. EEG Data Recording & Analysis

EEG data were recorded from a 64-channel active BioSemi system with Ag/AgCl electrodes placed according to the international 10/20 system fitted to the participant’s head. Six external electrodes were used: four to record eye movements (above and below the right, on the left and right temple), and two for offline referencing (left and right mastoid). Data were recorded at a 512 Hz sampling rate and we made sure the electrode offset was kept below 50 mV.

Data pre-processing and analysis used EEGLAB [58] and ERPLAB [59]. First, data points were re-referenced to the average of the right and left mastoids. They were then bandpass-filtered with half power cut-offs at 0.1 and 30 Hz at 12 dB/octave. Time windows from 100 to 600 ms post stimulus onset were extracted (“epoched”) from the EEG signal and baseline-corrected by subtracting the mean voltage in the 100 ms pre-stimulus interval from each sample in the window. Independent component analyses were conducted to identify and remove noisy EEG channels. Eye-movement components based on the activity power spectrum, scalp topography, and activity over trials were also removed. Noisy EEG channels that were removed were interpolated using spherical spline interpolation. Artefacts above 70 mV were rejected automatically for all channels. Participants with more than 40% of artefact-contaminated epochs were excluded from further analyses (n = 6). The epochs were averaged separately for standards (excluding the first 20 standards and the standards immediately following a deviant stimulus), for each deviant token, and for each control block.

Two difference waves were calculated by subtracting the mean ERP response to each control stimulus from the mean ERP response to its deviant counterpart. These difference waves were then grand-averaged across participants. In the grand-averaged waveform, we sought a negative peak 100 to 250 ms after consonant production (taking the 20 ms consonant portion of the stimulus into consideration) to ensure that we were measuring the neural response to the tone. This resulted in measuring the 120 to 270 ms time window post-stimulus onset. We then centred a 40 ms time window at the identified peak and measured the mean amplitude in that window per individual participant (cf. [47]). These mean individual amplitudes were our measure of MMN amplitude in further statistical analyses. Within the same 40 ms time window, latency was measured by establishing the most negative peak for each participant. These mean individual latencies then became the measure of MMN latency in the further analyses.

### 3.2. Results

Following previous studies (e.g., [47,60]), MMN amplitudes and latencies were measured at nine channels (Fz, FCz, Cz, F3, FC3, C3, F4, FC4, C4) and were analysed in two separate mixed analyses of variance (ANOVAs) with a between-subject factor of condition (unimodal vs. bimodal) and within-subject factors of phase (pre- vs. post-training), anteriority (Frontal (F) vs. frontocentral (FC) vs. central I), and laterality (left, middle, right). Based on activating different neural populations, the dependent variables including mean amplitude and peak latency may reflect different processing mechanisms [61]: the former may show the robustness of listeners’ discrimination as well as the acoustic/phonetic difference between the standard and the deviant stimuli, and the latter may reflect the required time to process the difference between the stimuli. Both variables have been used as auditory perceptual processing measures at early pre-attentive levels for native and non-native speech [36,62]. MMN responses tend to occur at frontal (F) and frontocentral (FC) sites. If distributional training has an effect on tone perception, we predicted an increase in MMN amplitude at those sites between pre- and post-test, as evidence of the auditory change in stimuli initiating an involuntary attentional switch [39,63].

**Figure 5:**
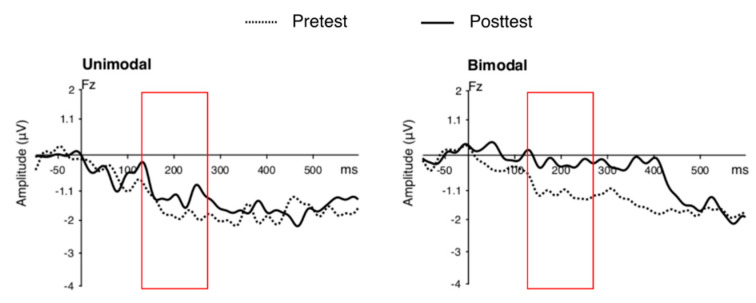
Grand-averaged MMN component by unimodal (**left**) and bimodal (**right**) condition. Dotted lines show the MMN component at pre-training and solid lines represent the MMN component at post-training. The red boxes highlight the time window in which the MMN amplitude peaks were measured (i.e., 120–270 ms post-stimulus onset to account for consonant production).

*MMN mean amplitude.* Figure 5 shows the grand-averaged MMN component in response to the contrast at pre- and post-training. There was a main effect of phase (*F*(1, 30) = 4.37, *p* = 0.045, *η*_g_^2^ = 0.046). Specifically, the MMN amplitude at pre-test (*M* = −1.67, *SD* = 3.03) was larger than that at post-test (*M* = −0.80, *SD* = 2.56). No other effects or interactions were significant.

**Figure 6:**
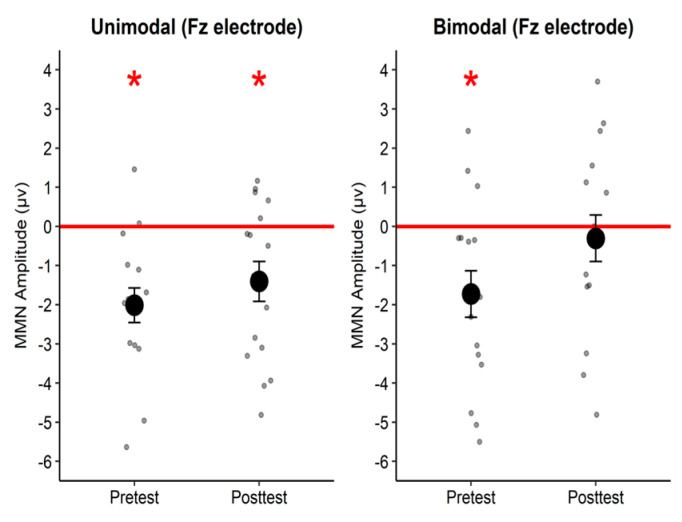
Mean MMN amplitude (large dots) for the two conditions at each test phase. Small dots represent individual data. Error bars represent one standard error. Asterisks represent significant MMN amplitude.

As condition was our variable of interest, the MMN amplitude response at the Fz electrode site in each phase was compared against zero (Figure 6) following previous literature [49,60,61,64,65]. Participants in the unimodal group exhibited significant MMN amplitudes at pre-test (*t*(15) = −4.55, *p* < 0.001, *d* = −1.14) and at post-test (*t*(15) = −2.76, *p* = 0.015, *d* = −0.69), whereas participants in the bimodal group exhibited a significant MMN amplitude at pre-test (*t*(15) = −2.90, *p* = 0.011, *d* = −0.72) but not at post-test (*t*(15) = −0.51, *p* = 0.616, *d* = −0.13). Paired t-tests comparing the MMN amplitude between pre- and post-test for each condition revealed no difference for the unimodal group (*t*(15) = −1.01, *p* = 0.329, *d* = −0.23) whereas there was a marginal difference for the bimodal group (*t*(15) = −2.12, *p* = 0.051, *d* = −0.35), indicating statistical-learning-induced changes.

*MMN peak latency.* A mixed ANOVA with condition (2-level, unimodal vs. bimodal) as the between-subjects factor, and within-subject factors of test (pre- vs. post-test), anteriority (F vs. FC vs. C), and laterality (left vs. middle vs. right) was conducted on the mean MMN peak latency. Main effects of phase (*F*(1, 30) = 240.35, *p* < 0.001, *η*_g_^2^ = 0.729) and condition (*F*(1, 30) = 24.57, *p* < 0.001, *η*_g_^2^ = 0.179) were found, which were qualified by a significant phase × condition interaction (*F*(1, 30) = 19.68, *p* < 0.001, *η*_g_^2^ = 0.177). Post hoc Tukey tests revealed that there were no group differences in latency at pre-test (*M*_diff_ = 0.05, *p* = 0.986) but at post-test, participants in the bimodal group had significantly earlier MMN peaks than those in the unimodal group (*M*_diff_ = 20.39, *p* < 0.001). No other effects nor interactions were significant.

### 3.3. Discussion

Experiment 2 showed learning-induced neurophysiological changes in these listeners’ tone perception at pre- and post-test. Reduction in tonal sensitivity was significant in the bimodal condition. In addition, the latency results showed an earlier peak in the post- than in the pre-test, with a larger impact again in the bimodal condition. Although behavioural results indicated limited discrimination prior to training, neural sensitivity was consistent with discrimination. Note that the pre-test sensitivity is not unexpected given listeners’ psycho-acoustic sensitivity to non-native tones across ages [51], with sensitivity modulated by tone salience [66].

At post-test, although behavioural outcomes indicated improved discrimination, listeners’ neural processing was generally weakened in both conditions. The results from the bimodal distribution may suggest that the increased exposure and familiarity with tones in the neural experiment hindered distributional learning. Alternatively, although the acoustic experience had given listeners more familiarity with tones, the information they received was insufficient to establish tonal categories from the frequency distribution.

Indeed, in the bimodal condition where MMN responses were diminished, frequency peaks in the distribution were near the two ends of the continuum (Figure 2, steps 2–7), whereas the peaks in the frequency distribution were at the midpoint (Figure 2, steps 4–5) in the unimodal condition. To process stimuli efficiently, listeners may focus on the most frequently presented (hence, most salient) stimuli in each condition. In the bimodal condition, steps 2 and 7 were highlighted when played alongside stimuli close to the discrimination boundary (steps 3 and 6), and listeners may expect a similar (or larger) difference to detect deviation compared with post-test. Contrasts with a smaller acoustic difference (i.e., steps 3–6) may then be harder to detect. On the other hand, those who were exposed to the unimodal condition, where steps 4 and 5 were the most frequent, would show neural responses to a contrast of larger acoustic difference (i.e., steps 3–6). In other words, the most frequent and prominent steps, namely the peaks of each distribution, would impact subsequent perception (To explore our proposed hypothesis on the impact of frequency peak, an additional behavioural test was conducted measuring Australian listeners’ (*N* = 12) sensitivity to the designated tonal contrasts with manipulations of the acoustic distance between the tokens. While discrimination of steps 2–7, the acoustically distant contrast mostly presented in the bimodal condition, reached 82% accuracy, discrimination of steps 3–6 was at a chance (*p* = 0.511 against 0.5) and accuracy in the discrimination of steps 4–5, the acoustically close contrast presented most frequently in the unimodal condition, was 20%. These results reflect contrast salience). The overall pattern suggests that listeners’ sensitivity is more auditory or psychophysical than phonetic or phonological at this stage: they can discriminate, but fail to establish categories.

With either an explanation in terms of acoustic salience, or an explanation in terms of perceptual assimilation, certain interactions between acoustic and statistical cues in listeners’ neural processing will be assumed. Previous research has not only shown humans’ ability to track input frequency distributions from the ambient environment, and their ability to abstract and retain the memory of non-native pitch directional cues, but also clear ability to shift their weighting of acoustic/phonetic cues and to reconfigure their learning strategies [19,20]. In a speech segmentation task when both statistical and prosodic cues are presented, listeners attend more to the latter to acquire speech information [21]. Indeed, when various types of cues (e.g., acoustic feature, frequency distribution) are presented to listeners, the weighting of these cues may be dynamic and change in real-time during experimental training [67,68]. Last but not least, the outcomes also confirm that EEG is more sensitive than behavioral measures in revealing listeners’ responses in the course of speech perception [34,35,36,37].

## 4. General Discussion

This study tested the learning of a non-native tone contrast by non-tonal language speakers. Data were collected with both behavioural and neural test methods, statistical distributions were varied (along a single continuum) in the input, and both identification and discrimination data were collected and analysed. Non-native listeners’ tone discrimination improved after both unimodal and bimodal exposure, and there was a reduction in sensitivity after training (observed with the more sensitive measures, to wit, MMN amplitude and latency, which were recorded using EEG). In the EEG data, perceptual differences emerged between the two exposure conditions. The bimodal condition appears to have a more negative impact than the unimodal condition after training. The reduced amplitude and early latency in the bimodal condition may reflect inability to establish categories from the frequency distribution, although listeners had become more familiar with tones after training.

Our neurophysiological finding contrasts with results in prior distributional learning studies (see Figure 2), where a bimodal distribution leads listeners to display enhanced distinction of steps midway along the distribution, while theeffect is reversed with the unimodal distribution, where steps within one peak become less distinct perceptually. We proposed that the lack of sensitivity at post-test after bimodal training is due to the exposure to a salient contrast during training that participants then expected to find again at test. In the absence of distributional learning, there was exploitation of salient acoustic cues instead. This interpretation is in line with the phonetic-magnitude hypothesis [44], which holds that the size of acoustic features (or phonetic distinctions, articulatory correlates) plays a more central role than the extent of native language experience, an interpretation consistent with the Second-Language Linguistic Perception model (L2LP; [69,70,71,72]), which highlights the interaction between listeners’ native phonology and the magnitude of the phonetic difference in auditory dimensions. In a way, the adult listeners in the current study behaved like infants without prior knowledge of lexical tones and like adult L2 learners without prior knowledge of vowel duration contrasts. They concentrated on the most salient phonetic cues, while ignoring or lowering the weighting of other cues.

We further argue that compared to segmental features, pitch is a particularly salient feature. Pitch contrasts, regardless of height [41,42] or direction (as illustrated in the current study), may be more prone to be weighted acoustically compared to statistically, especially among non-tone language speakers who predominantly use pitch in pragmatic but not lexical functions. Note that this finding was only seen neurally and not behaviourally, which speaks to the fact that this might only be detected through the deployment of sensitive measures.

When native listeners process lexical tones, they attend to linguistic features such as pitch, intensity, and duration, based on their existing knowledge of the categorical structure and the phonotactics of their language [73]. When non-native listeners are presented with the same input, they have no relevant categorical or phonotactic knowledge to call upon. But their auditory abilities may be assumed to parallel those of the native listener, so that it is not surprising that simple discrimination elicits a similar pattern of performance for native and non-native listeners [25], even when any task involving recourse to frequency distributions leads to very different results from these two listener groups.

Results conform to a heuristic approach to processing: when hearing a subtle non-native tone contrast, listeners’ neural sensitivity may be insufficient to perceive the fine-grained tone steps and turn more towards acoustic information, and especially the most frequently presented (i.e., most salient) stimuli within each type of distribution. The general pattern somewhat conforms to previous findings showing that a bimodal distribution does not necessarily promote distinct discrimination between the two peaks [13,38,39], and a unimodal distribution does not always hinder it [43]. Factors such as acoustic properties or perceptual salience between tokens may play a role.

Furthermore, we argue that listeners who were trained on a bimodal distribution in which the peak contrast (steps 2–7) contains a large, discriminable acoustic distance may exhibit hampered discrimination of contrasts that rest on smaller differences. In consequence, smaller differences along the continuum may be disregarded. On the other hand, training on a unimodal distribution in which the peak contrast (steps 4–5) is extremely difficult to discriminate may ease the processing of contrasts with a larger acoustic difference (i.e., steps 3 and 6). This explanation does not come out of the blue, as similar ideas have been proposed for studies showing that infants are sensitive to acoustically subtle phonetic contrasts [74] and develop their word learning abilities of very small differences in vowels in a speedy fashion [75]. This interpretation assumes that listener sensitivity to ambient acoustic and statistical information can pave the way for perception and learning of new contrasts in a second language. However, our results should not be construed as evidence that native English speakers cannot achieve a level of tone categorization matching that of native tone speakers [76]. Many factors may play a role. For example, past research has shown that although statistical information can prompt the formation of distinct phonemic categories within three minutes for some non-native contrasts, longer duration of exposure may be required to trigger learning in a bimodal condition [13]. In a previous study examining listeners’ non-native tone discrimination ability, an overall effect of learning surfaced after around ten minutes of exposure [52]. In the present experiment, embedded statistical information altered perception within six minutes of exposure. The take-home message for this study is that for non-native contrasts presented without context, the acoustic salience of the sounds may initially matter more than the statistical distribution of the sounds. At least in the initial stages of learning, distributional evidence may play only a minor role. Future studies can investigate whether increased input might introduce robust effects of exposure to a bimodal distribution.

## Figures and Tables

**Table 1:** Mean (SD) accuracy percentage and corresponding t and *p* values in one-sample t-test against the chance level in bimodal and unimodal before and after distributional learning in the discrimination task. (See Supplementary Material C for descriptive statistics of other contrasts.).

		Mean	SD	T	*p*
Bimodal	Pre	60.83%	35.62%	1.490	0.150
	Post	67.50%	33.26%	2.577	0.017
Unimodal	Pre	51.67%	30.59%	0.267	0.792
	Post	65.00%	30.21%	2.432	0.023

**Table 2:** Mean (SD) percentage of choosing falling over flat tones in bimodal and unimodal before and after distributional learning in the identification task. (See Supplementary Material D for descriptive statistics of other contrasts.).

		Step 3				Step 6			
		Mean	SD	t	*p*	Mean	SD	t	*p*
Bimodal	Pre	34.75%	28.62%	−2.610	0.016	67.29%	31.61%	2.680	0.013
	Post	25.08%	33.30%	−3.665	0.001	65.87%	35.28%	2.204	0.038
Unimodal	Pre	45.83%	28.30%	−0.721	0.478	74.29%	29.79%	3.994	0.001
	Post	42.25%	28.98%	−1.310	0.203	69.38%	29.81%	3.183	0.004

## Data Availability

Anonymized data is available upon request to the first author.

## Supplementary Materials

The following supporting information can be downloaded at: https://www.mdpi.com/article/10.3390/brainsci12050559/s1, Supplementary Material A: F0 values at 4 points along the /taT1/-/taT4/ continuum; Supplementary Material B: Pitch-Contour Perception Test results; Supplementary Material C: Discrimination task results across all contrasts; Supplementary Material D: Identification task results across all steps.
