# Supplementary material for: Learning to Perceive Non-Native Tones via Distributional Training: Effects of Task and Acoustic Cue Weighting
Source: Brain Sci. 2022 Apr 27;12(5):559. doi: 10.3390/brainsci12050559 (PMC9138676; doi:10.3390/brainsci12050559)
Supplement: Supplementary file 1 [file brainsci-12-00559-s001.zip › brainsci-1646460-supplementary.pdf]

brain sciences

MDPI

Supplementary Material A

F0 values at 4 points along the /taT1/-/taT4/ continuum. In the table, /taT1/ = stimulus 1 and /taT4/ = stimulus 8. The intermediate numbers correspond to the stimuli created along the continua.

Step	Starting Point	Interpolate 1	Interpolate 2	Ending Point
1	278.795	276.670	274.525	272.390
2	293.044	284.788	259.178	255.264
3	307.293	292.906	243.829	238.136
4	321.542	301.024	228.480	221.008
5	335.791	309.142	213.131	203.880
6	350.040	317.260	197.782	186.752
7	364.289	325.378	182.433	169.624
8	378.537	333.494	167.084	152.496

Supplementary Material B

As an additional control, the Pitch-Contour Perception Test (PCPT; [1,2]) was included in the post-training phase, to examine participants' pitch perceptual abilities. This test required indication of whether isolated tone tokens had a flat, rising or falling contour. The PCPT allows division of participants into high and low aptitude groups, to examine whether ability to perceive pitch affects identification and discrimination responses. No differences either in the identification or in the discrimination tasks were observed between listeners with high versus low aptitude in the present study.

1. Wong, P. C.; errachione, T. K. Learning pitch patterns in lexical identification by native English-speaking adults. Applied *Psycholinguistics* **2007**, *28*(4), 565-585.

2. Perrachione, T. K.; Lee, J.; Ha, L. Y.; Wong, P. C. Learning a novel phonological contrast depends on interactions between individual differences and training paradigm design. *The Journal of the Acoustical Society of America* **2011**, *130*(1), 461-472.

Supplementary Material C

The table below represents the discrimination task results across all contrasts. Each grid represents the mean (SD) percentage of accuracy before ("Pre") and after ("Post") bimodal or unimodal exposure.

		Contrasts							
		1-1	1-3	2-2	2-4	3-3	3-5	3-6	4-4
Bimodal	Pre	95.40%(10.29%)	84.60%(21.40%)	92.30%(15.05%)	59.20%(29.11%)	90.80%(15.21%)	50.00%(30.59%)	60.83%(35.62%)	93.10%(12.58%)
	Post	97.70%(06.52%)	90.00%(23.49%)	96.20%(09.83%)	61.50%(36.63%)	93.80%(12.35%)	46.20%(34.30%)	67.50%(33.26%)	93.80%(13.59%)
Unimodal	Pre	91.50%(22.03%)	87.70%(20.46%)	90.00%(17.20%)	50.00%(31.11%)	89.20%(18.09%)	41.50%(29.35%)	51.67%(30.59%)	86.90%(23.28%)
	Post	95.40%(11.74%)	96.20%(09.83%)	93.10%(20.35%)	63.80%(33.48%)	93.80%(13.59%)	58.50%(35.74%)	65.00%(30.21%)	90.00%(22.09%)

		Contrasts						
		4-6	5-5	5-7	6-6	6-8	7-7	8-8
Bimodal	Pre	48.50%(38.02%)	96.20%(09.83%)	50.80%(32.61%)	91.50%(15.15%)	37.70%(34.56%)	95.40%(14.21%)	94.60%(09.05%)
	Post	45.40%(33.25%)	94.60%(12.08%)	52.30%(35.36%)	86.20%(23.16%)	49.20%(34.98%)	95.40%(11.74%)	94.60%(14.49%)
Unimodal	Pre	40.80%(33.70%)	94.60%(16.55%)	40.00%(34.41%)	93.10%(18.71%)	39.20%(35.99%)	90.80%(15.21%)	91.50%(18.91%)
	Post	43.80%(33.48%)	91.50%(18.04%)	50.00%(36.33%)	89.20%(22.08%)	43.80%(31.51%)	89.20%(23.48%)	92.30%(21.22%)

Supplementary Material D

The table below represents the identification task results across all steps. Each grid represents the mean (SD) proportion of choosing "falling" over "flat" before ("Pre") and after ("Post") bimodal or unimodal exposure.

		Steps							
		1	2	3	4	5	6	7	8
Bimodal	Pre	41.66%(35.97%)	32.05%(31.59%)	34.75%(28.62%)	42.94%(31.33%)	52.56%(35.49%)	67.29%(31.61%)	68.58%(32.43%)	64.74%(37.51%)
	Post	44.87%(38.80%)	26.92%(31.29%)	25.08%(33.30%)	42.30%(34.06%)	54.48%(39.03%)	65.87%(35.28%)	68.58%(36.61%)	64.74%(36.91%)
Unimodal	Pre	40.38%(35.95%)	26.92%(24.97%)	45.83%(28.30%)	55.12%(33.58%)	57.05%(34.37%)	74.29%(29.79%)	70.51%(32.76%)	78.20%(28.97%)
	Post	42.94%(35.33%)	22.43%(28.26%)	42.25%(28.98%)	52.56%(35.49%)	55.76%(35.88%)	69.38%(29.81%)	62.82%(32.07%)	69.87%(29.06%)
